# Identifying suitable mussel cultivation sites in European offshore waters—an assessment for co-location with the wind industry

**DOI:** 10.1038/s44183-026-00187-0

**Published:** 2026-02-27

**Authors:** Enora M. Lecordier, Pierre Gernez, Krysia Mazik, Ethan Clark, Rodney M. Forster

**Affiliations:** 1 Energy and Environment Institute, University of Hull, Hull, UK; 2 Nantes Université, Institut des Substances et Organismes de la Mer, ISOMER, UR 2160, Nantes, France; 3 School of Environmental and Life Sciences, University of Hull, Hull, UK; 4 The Dove Marine Laboratory, School of Natural and Environmental Sciences, Newcastle University, Newcastle-upon-Tyne, UK; 5 Hull Marine Laboratory, School of Environmental and Life Sciences, University of Hull, Hull, UK

**Keywords:** Climate sciences, Ecology, Ecology, Environmental sciences, Ocean sciences

## Abstract

Ensuring food security is a vital challenge. To meet food and, especially, protein demand in the next few decades, the aquaculture industry needs to expand. This could be achieved by expanding marine aquaculture at sea. Moving aquaculture plots further offshore has gained interest due to its increased space availability and more stable conditions compared to coastal areas, while also mitigating the effects of climate change extremes inshore. Spatial multi-criteria evaluation allowed for the identification of regions in offshore European waters that, under present-day conditions, were both feasible and suitable for mussel cultivation (*Mytilus edulis* L.). Future climate models were also used and showed a latitudinal trend, making Northern European waters more suitable in the future, while the Southern part of Europe became too warm. However, the future impact of extreme events, such as marine heatwaves, is difficult to predict. In addition, the study identified offshore wind farms with potential for co-location with mussel cultivation, which could help concentrate human uses at sea and reduce the extent of marine areas subject to anthropogenic pressure. With the offshore wind industry expanding rapidly in the future, even more co-location options will become possible.

## Introduction

Ensuring food security and improving nutrition are immense, vital and contemporary challenges, as evidenced by the United Nations “Zero Hunger” goal to create a world free of hunger by 2030. In 2019, the consumption of aquatic food accounted for 17% of animal protein intake worldwide with 21.1 kg/capita/year^1^. According to Fukase & Martin^2^, the worldwide food demand will increase more rapidly than the food supply by 2050, meaning that sustainable edible seafood yield would need to increase by 36 to 74% to meet the 2050 food demand^3^. Seafood, including bivalve molluscs, has high nutritional value, has the potential to reduce meat consumption and has a significantly lower environmental footprint than meat production^4^. Therefore, seafood consumption could enhance protein and nutrient intake in populations where deficiencies are prevalent.

The Mytilidae (mussel) family is the fourth most cultivated bivalve worldwide after oysters, clams, and scallops^1^. It has been considered a healthy food source and sustainable over time, with a bivalve mollusc industry worth USD 4.3 billion in 2020^5^. Despite nutritional and economic benefits, the mussel cultivation industry has seen slow growth in the past decades^1,6^. This could be due to limited space at sea (restricted by coastal water quality, among other reasons) and dominance of the sector by relatively small companies unwilling or unable to risk expanding^7^. Traditionally, mussels are grown on poles (*bouchots*), on suspended ropes (longlines and rafts), or as bottom culture^8^. Smaal^8,9^ described future scenarios for cultivation and recognised that if the mussel industry was to grow, questions such as finding new areas for cultivation, assessing the environmental impact of such expansion and opening collaboration with other stakeholders needed to be investigated.

The impact of climate change also leads to higher risks for cultivation. Indeed, the prevalence of terrestrial and marine heatwaves is likely to alter biogeochemical regimes to a higher degree nearshore than offshore^10^. In coastal cultivation plots, high seawater temperatures were shown to alter mussel development as they induced physiological stress and increased the risk of disease^11^. As another example, massive intertidal mussel bed fatalities were experienced in Canada following 3 days of extreme weather in 2021^12^. Kamermans & Saurel^13^ showed that a population of *Mytilus edulis L*. began to experience the death of organisms after 3 to 5 days in lab cultivation when water exceeded 25 °C.

These lead to the consideration of open ocean culture, which is becoming increasingly attractive for the industry because of its spatial development potential and stable conditions. Offshore waters may present fewer contaminants (being further away from land sources and allowing for higher dilution) and a reduced risk of proliferation of toxic algae and disease outbreaks that could damage the cultivated stocks^14–17^. Compared to intertidal areas, the open ocean provides complete immersion time for mussels (thus reducing exposure to extreme temperatures and increasing time to feed) and, with its more stable environmental conditions, minimises fluctuations in salinity, both of which support more rapid growth^18^.

Offshore cultivation seems to be feasible and to offer a better yield than nearshore cultivation. However, very few potential offshore areas for aquaculture have been exploited because of the economic costs and safety aspects of working in remote areas at sea, where larger and more robust infrastructures and logistics are required due to high waves and strong current regimes^19^. From a technical perspective, wave height is an important component of offshore aquaculture, as too high waves can damage the structure, especially the anchoring of the longlines^20^. There is evidence of mussel farms operating under nearly 10 m maximum wave height^21^. Nevertheless, even though polypropylene lines can resist 6 m wave height and current regimes of up to 1.5 m s^−1^, it seems to be the upper limit for steel hawser-based line cultivation method^22^.

Also, the wave effect is highly dependent on the cultivation system type, as longlines placed deeper than 5 m are generally spared by the highest waves^23^. Thus, the effect of wave height and current speed on cultivation lines is highly dependent on the type of line and the depth of the cultivation. High waves can also increase safety risk for operators at sea. For example, offshore wind (OSW) technicians’ health and safety limit for operating at sea is usually 1.5 m for small-sized vessels^24–26^. It seems plausible that an offshore farming vessel would operate under the same limitations. For the required water depth, offshore aquaculture can be practised up to 100 m depth^27^. From an economic perspective, the upper limit for aquaculture feasibility in terms of distance from port was found to be 25 nm^27^, even though some nearshore cultivation seemed to be up to 55 nm from the nearest port^28^.

Those technical and economic requirements, added to the yield uncertainty due to a lack of data, might make some mussel farmers, already established nearshore, reluctant to expand further out. In addition, expanding anthropogenic activities at sea (such as offshore wind parks) are causing a reduction of space availability and conflicts between stakeholders. A way to overcome those challenges is through marine spatial planning (MSP), to allocate shared space at sea, underpinned by scientific knowledge and predictions.

**Fig. 1:**
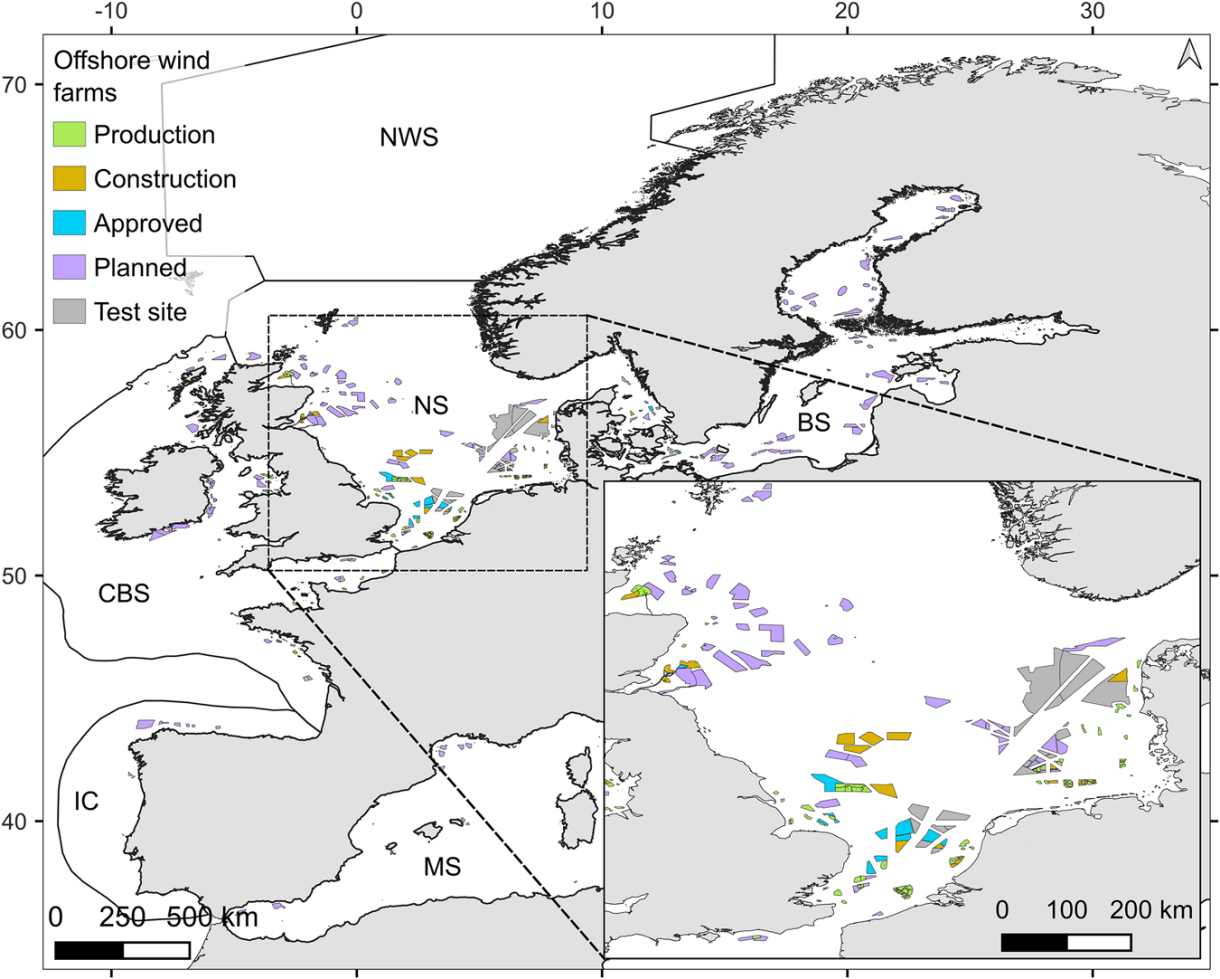
Status of offshore wind farms in Europe in 2024. Offshore wind farms at different stages of development in Europe are shown (data adapted from EMODnet^102^). Large Marine Ecosystems are presented: NWS Norwegian Sea, NS North Sea, BS Baltic Sea, CBS Celtic-Biscay Shelf, IC Iberian Coastal, MS Mediterranean Sea.

Another sector, the OSW industry, has shown a constantly increasing footprint on maritime space and its management in the past decade (Fig. 1). The OSW industry needs a significant space at sea to achieve the United Nations carbon emission 2050 target, and thus, pressure on maritime space will continue to increase, raising even more conflicts between stakeholders^29,30^. These emerging conflicts have increased interest in MSP and policy-making, and the opportunity for co-existence still requires investigation^31^. Co-existence implies that two or more anthropogenic activities share the space. As this concept depicts a continuous interaction between stakeholders, a variety of typologies exist depending on the degree of synergy shared^32^. For instance, under the co-existence umbrella, “co-location” is the interaction where two or more activities use the same space at the same time, with neutral benefits for each side^31^. This is different from “multi-use”, where activities use the same space, at the same time, but also share infrastructures and services, ideally, with benefits for each side. These two terms, being both MSP methods, are often used interchangeably even though they imply different levels of co-existence. It was shown that a robust framework and regulations are still needed to facilitate sharing space at sea and for co-existence to be sustainable for industries^19,33^. This study mainly focuses on the co-location potential, but some insights are also useful for the multi-use concept.

The aquaculture industry could benefit from co-location with the OSW industry as a facilitator to expand into the open sea. Indeed, the OSW industry is already well established in Europe and experienced in accessing remote areas. OSW structures are robust and resistant to high-energy environments, and due to the size of the investments, wind farms usually have detailed scientific information available at the site level. The assessment of the potential for co-existence between aquaculture and OSW was part of the focus of many small-scale MSP research trials and conceptual studies, in particular, the projects MERMAID, TROPOS, and MUSES^7,34–38^. These projects investigated the infrastructural needs for multi-use at sea and the state of co-location development in Europe. They are now a reference in co-existence project development, mainly between the OSW industry and other maritime stakeholders. They helped increase awareness about co-location and multi-use opportunities and recommendations for the future.

More specifically, co-locating mussel aquaculture with the OSW industry has been investigated in various ways, such as environmental suitability, stakeholders’ needs and benefits^33,39,40^ and economic viability^23,41,42^. Aquaculture site identification and planning have been carried out in the past using spatial multi-criteria evaluation (SMCE)^43–54^. SMCE is a decision-making process that combines factor analysis and spatial data processing to study and prioritise potential solutions and scenarios^55^. This method can quickly process new assets and give interpretable outcomes for policymakers^56^.

In this study, a SMCE was developed to identify the optimal location for *M. edulis* offshore aquaculture in Europe. There are different ways to handle the underlying spatial data layers in an SMCE: using Boolean (optimal/not-suitable) or fuzzy logic (sometimes called the “fuzzy method”) which involves fitting a mathematical function over each factor^18,57,58^. It was shown that most of the applied GIS studies for aquaculture focused on small-scale areas in a single country, and only a few drew a big picture for aquaculture on regional and global scales, usually used for assessment and trend scenarios^59^. Here, using a SMCE approach, we focus on European waters and identify feasible and suitable areas for implementing *M. edulis* cultivation offshore, from both technical and biological perspectives. In addition, the paper identifies potential OSW farms that could be used for offshore mussel aquaculture as well. Consideration is also given to the suitability of mussel cultivation offshore by 2050 with respect to climate change (e.g. warming seas) and future OSW expansion scenarios. However, this study does not consider socio-economic considerations such as operational costs, job creation, spat supply, or yield estimates.

## Results

**Fig. 2:**
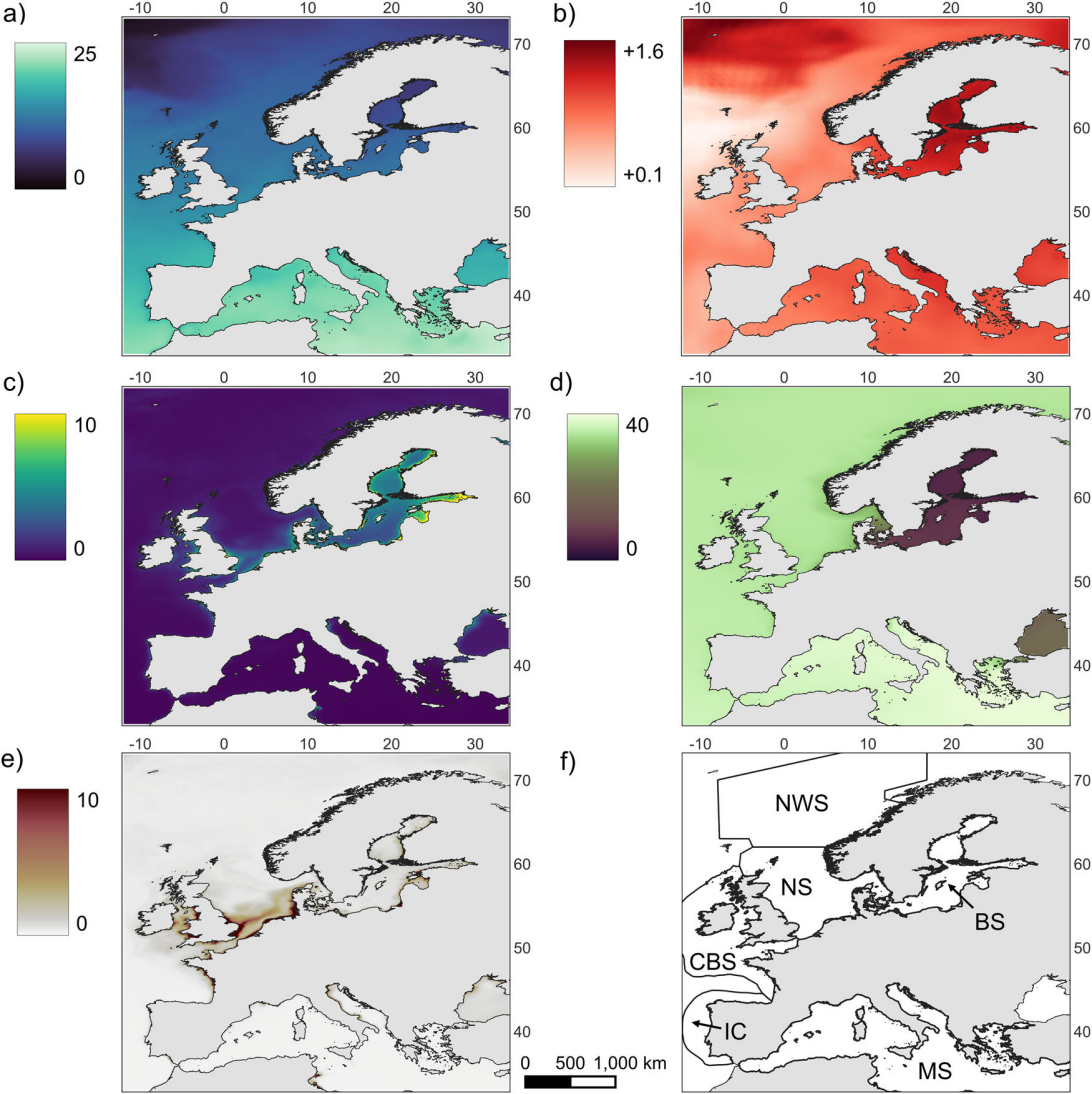
Mean annual oceanic conditions (2019–2023) and future projections (2040–2050) for large marine ecosystems (LME) in Europe. Mean annual **a** sea surface temperature (°C), **c** chlorophyll a concentration (mg.m^−3^), **d** salinity, and **e** suspended particulate inorganic matter (g.m^−3^) for 2019–2023. **b** Projected increase in mean sea surface temperature (°C) by 2040–2050 under the SSP5-8.5 scenario. **f** Studied large marine ecosystems (LME).

In European waters, the yearly average sea surface temperature (SST) data were highly variable, from 7 °C in the Baltic Sea to 24 °C in the Mediterranean Sea, following a latitudinal pattern (Fig. 2a). The SSP5-8.5 scenarios predicted a global warming of European waters by 2050 going from +0.1 °C off the coast of Northern Ireland and Scotland to +1.6 °C at high latitudes and in the Baltic Sea (Fig. 2b). Chlorophyll a (CHL) concentration was generally more abundant in nearshore areas as well as in the North Sea and Baltic Sea (Fig. 2c). Salinity was much lower in the Baltic Sea and the Black Sea than in the rest of Europe, while the Mediterranean Sea showed the highest value (Fig. 2d). High suspended particulate inorganic matter (SPM) concentration was mainly observed nearshore, with hotspots off the main river outflows (Fig. 2e). To simplify the analysis, six Large Marine Ecosystems (LME) were studied (Fig. 2f): the Norwegian Sea (NWS), the North Sea (NS), the Baltic Sea (BS), the Celtic-Biscay Shelf (CBS), the Iberian Coastal (IC), and the Mediterranean Sea (MS).

### Feasibility

**Fig. 3:**
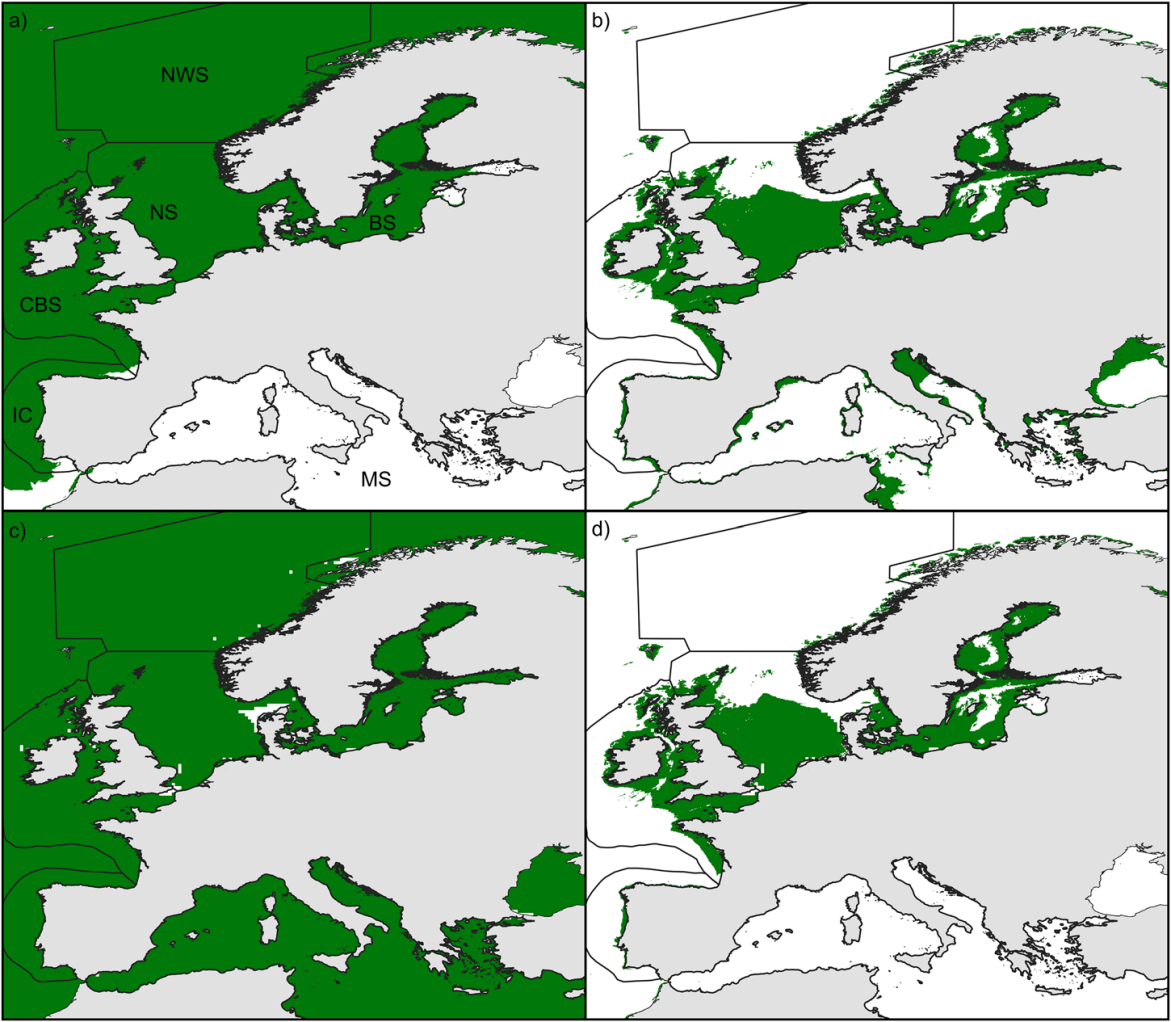
Feasibility masks for *M. edulis* cultivation derived from environmental constraints. Feasibility masks (in green) are presented based on **a** heat-spike occurrence, **b** bathymetry and **c** current speed. **d** shows the remaining feasible area for cultivation.

The feasibility study revealed heat spikes happening mostly in the Mediterranean Sea but also in the south of the Iberian Coastal and Celtic-Biscay Shelf waters, as well as in sheltered areas of the Baltic Sea (Fig. 3a). This geographical mask identified areas exceeding 25 °C for at least 3 consecutive days, ruling out areas of high risk of mussel mortality because of high SST episodes. The bathymetry was by far the most restrictive driver of the feasibility as it ruled out the largest area (Fig. 3b). The 1 m s^−1^ current speed limit was reached in highly dynamic areas such as the English Channel and the boundary between the North Sea and the Baltic Sea (Fig. 3c).

**Fig. 4:**
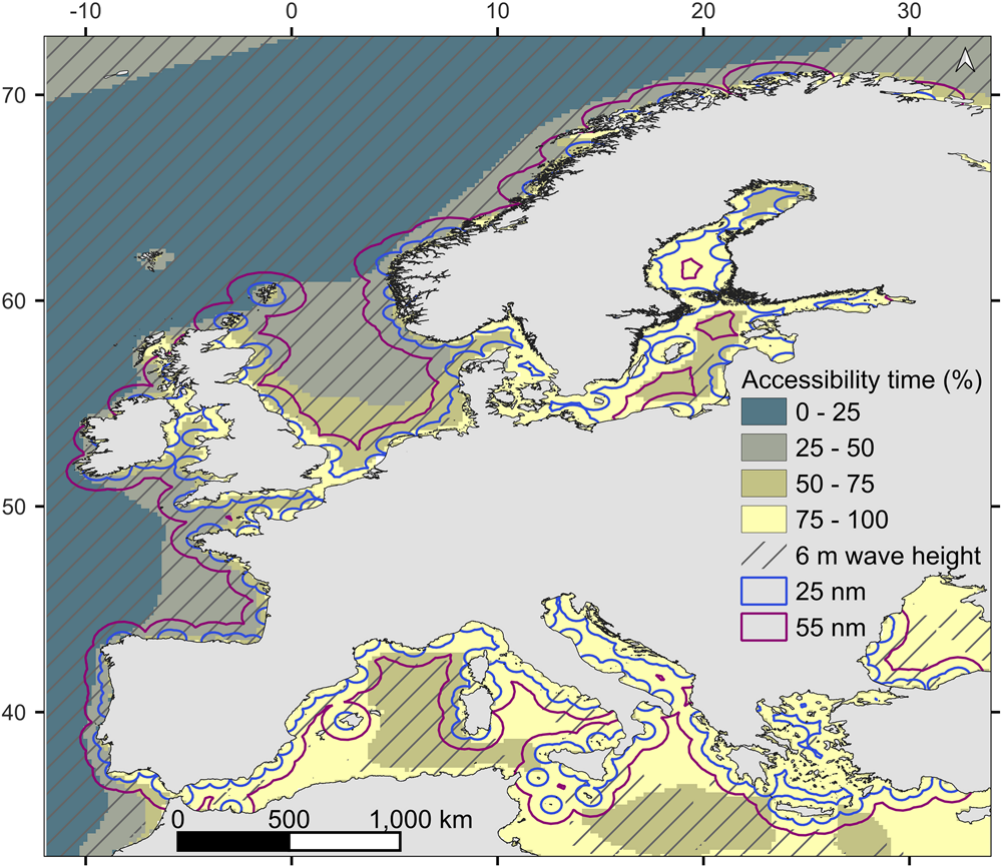
Additional exclusion criteria. To complement the spatial multi-criteria evaluation, other criteria can be used for decision-making, such as the Accessibility metric, maximum wave height and distance to port.

Overall, the potential for *M. edulis* cultivation in Europe was as high as 1,133,000 km^2^ (Fig. 3d), with the Baltic and North Seas presenting more than half of their surface identified as feasible (Table 1). Even though wave height and distance to the nearest port were not retained, in order to identify the broadly feasible areas, the accessibility metric, maximum wave height and distance to ports are presented in Fig. 4.

**Table 1:** LME area and relative area suitable for *M. edulis* cultivation

LME	Area in km^2^ (relative area, %)	LME	Area in km^2^ (relative area, %)
Baltic Sea (BS)	295,000 (75%)	Iberian Coastal (IC)	17,000 (6%)
North Sea (NS)	420,000 (60%)	Norwegian Sea (NWS)	19,000 (2%)
Celtic-Biscay Shelf (CBS)	326,000 (43%)	Mediterranean Sea (MS)	0 (0%)

A total of 420 out of the 454 OSW farms, from the planning stage to the operational stage, overlapped either completely or partially with the feasible area for mussel farming.

### Suitability

**Fig. 5:**
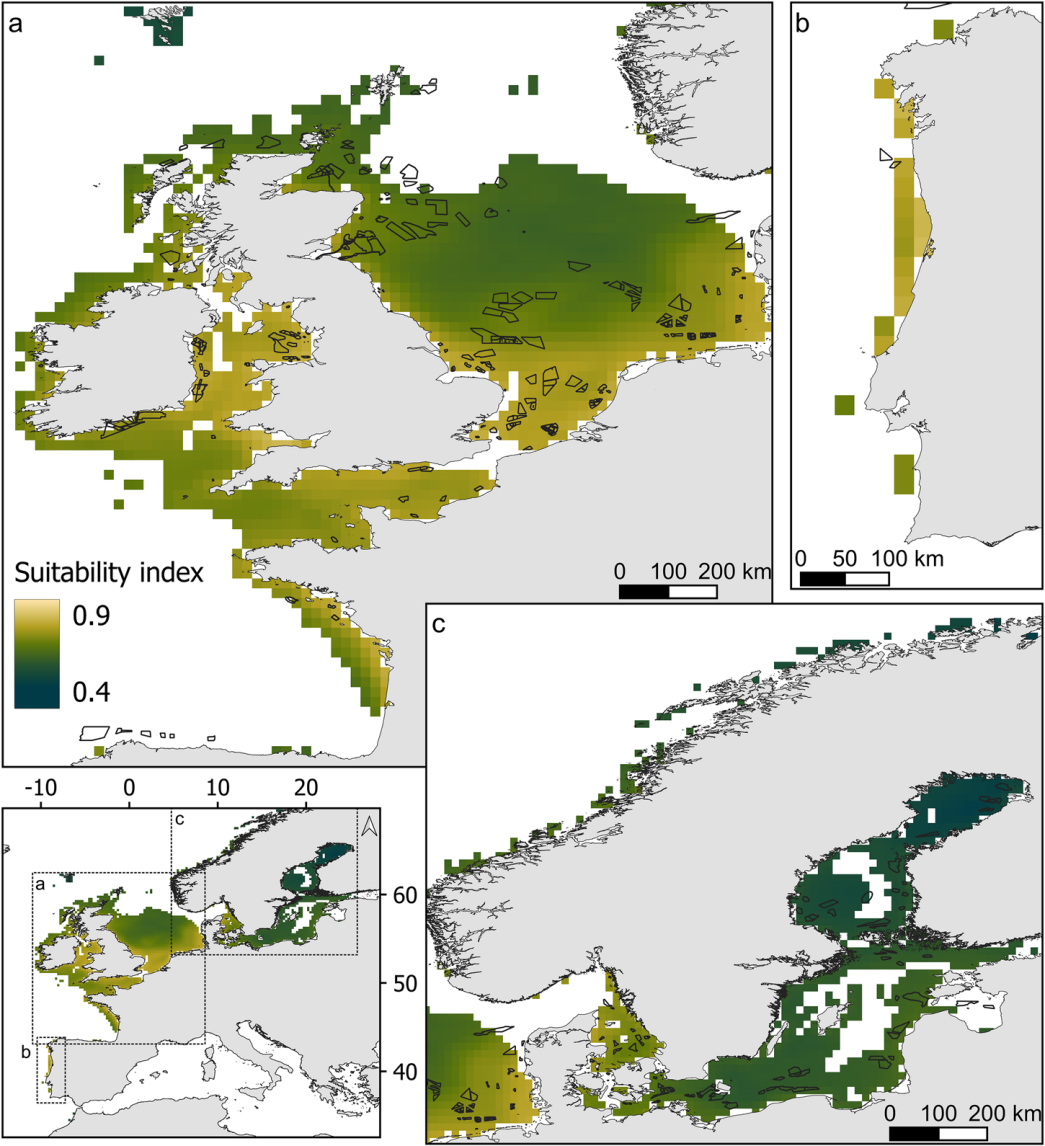
Suitability index for *M. edulis* cultivation in European offshore waters. The suitability index for areas identified as ‘feasible’ is shown for **a** the North Sea, the Celtic-Biscay Shelf, **b** the Iberian Coastal, and **c** the Baltic Sea. The black polygons represent offshore wind farm locations at different stages of development (in production, under construction, approved for construction, or planned). A suitability index of the full European extent can be found in the supplementary materials (Fig. S1).

**Fig. 6:**
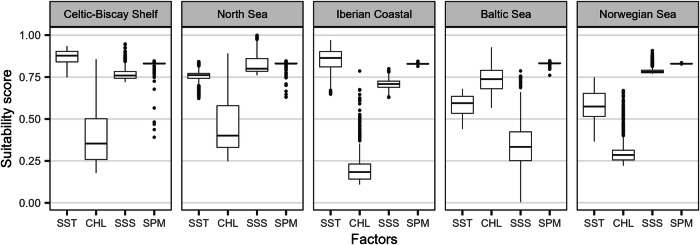
Suitability scores of environmental factors for *M. edulis* cultivation across large marine ecosystems (LME). The suitability scores across each LME are presented. SST sea surface temperature, CHL chlorophyll a concentration, SSS sea surface salinity, SPM suspended particulate inorganic matter concentration.

Approximately 185,000 km^2^ were found of medium suitability index (SI) (0.4–0.6), 1,010,000 km^2^ of high SI (0.6–0.8), and 4000 km^2^ of very high SI (> 0.8, Fig. 5). For the studied LME, a low CHL concentration was usually the main limiting factor, except for the Baltic Sea, for which the sea surface salinity (SSS) was the lowest (Fig. 6). The concentration of SPM was found to be of low importance in the suitability study.

In the Celtic-Bay Shelf, high SPM concentration could be found along the French and English coast with very high SPM concentration values above 50 g m^−3^ found off the River Severn (Fig. 2e). The highest SI was found in the Irish Sea and along the French coastline (Fig. 5). The SI ranged from 0.67 to 0.81 for the wind farms; the highest-ranked OSW was *Burbo Bank* in the Irish Sea. This site was accessible for more than 80% of the time, and at 6 nm, close to the nearest port (Table 2).

**Table 2:** Top-ranked wind farms for each large marine ecosystem

Large Marine Ecosystem	Wind farm	SI (± 2050 variation %)	Status	Area (km²)	Distance to port (nm)	Accessibility (%)
Celtic-Biscay Shelf	Burbo Bank (+ Extension)	0.81 (+2.68)	Pr	49.6	6.0	81.6
	Robin Rigg	0.80 (+2.77)	Pr	18.3	11.7	91.5
	Barrow	0.80 (+2.77)	Pr	10.0	5.9	81.6
	North Hoyle	0.80 (+2.69)	Pr	9.6	7.7	94.3
	Saint-Nazaire	0.80 (+1.07)	Pr	78.1	42	71.8
North Sea	Kentish Flats (+ Extension)	0.80 (+2.33)	Pr	18.2	12.8	99.0
	Humber Gateway	0.79 (+3.06)	Pr	26.5	15.7	90.1
	Race Bank	0.79 (+2.96)	Pr	62.4	34.3	81.8
	Inner Dowsing	0.79 (+2.93)	Pr	8.8	20.1	94.5
	Lynn	0.79 (+2.93)	Pr	7.9	18.6	94.5
Iberian Coastal	WindFloat Atlantic	0.79 (+0.19)	Pr	52.0	12.2	39.3
Baltic Sea	Lillebælt Syd	0.73 (+4.62)	Pl	54.2	6.0	79.9
	Omo Syd	0.72 (+4.84)	Pl	24.5	7.6	98.3
	Nysted	0.69 (+5.12)	Pr	23.1	5.8	99.6
	Rodsand II	0.69 (+5.09)	Pr	31.7	6.1	99.8
	Gennaker	0.68 (+5.46)	A	45.8	18.5	95.5

Only the top 5 wind farms are presented (fewer if there are less than 5 wind farms in the area). Wind farms are ranked by their average suitability index (SI) and then by Accessibility (%). The status of each wind farm is given (*Pr* production, *A* accepted, *Pl* planned).

In the southern part of the North Sea, the CHL concentration was generally high (Fig. 2c). It also presented high SPM concentration along the coast from Belgium to Denmark with values going up to 40 g m^−3^ due to the influence of British rivers (Fig. 2e). The SSS was mainly around 34–35, decreasing to 30 close to the coast of Norway, Denmark, and the Netherlands (Fig. 2d). The SSS dropped significantly as the Baltic Sea was approached. These combinations led to quite high suitability scores for each factor, with CHL being the main driver (Fig. 6). The highest SI was found in the southern part of the North Sea, mainly following the CHL gradient (Fig. 5). Within the wind farms, the SI varied from 0.65 to 0.80; the most suitable OSW farm was *Kentish Flats*, accessible 99% of the time and located at 13 nm from the nearest port (Table 2).

In the Iberian Coastal, SST was slightly lower along the coast compared to offshore at this latitude due to the upwelling effect bringing cold water into the area (Fig. 2a). Also, SPM and CHL concentrations were higher close to the shore than offshore but overall, very low in comparison with the North Sea (Fig. 2c, e). The CHL concentration was the limiting factor in the region for mussel growth (Fig. 6). Few suitable locations were found in the area, and these were mainly located along the coastline (Fig. 5). Only *Iberian WindFloat Atlantic* was found suitable with a SI of 0.79. This wind farm was found to be accessible only 39% of the time, at 12 nm from the nearest port (Table 2).

The SSS in the Baltic Sea was low, ranging from 3 to 15, which was found to be the limiting factor for *M. edulis* optimal growth (Figs. 2c, 6). Overall high CHL can be observed with values up to 7 mg m^−3^ on average in the most sheltered areas. The highest SI were found in the southern part of the Baltic Sea (Fig. 5). The wind farms in the Baltic Sea presented a SI ranging from 0.52 to 0.73; the highest ranked OSW farm being *Lillebælt Syd*, which was accessible 80% of the time at 6 nm from the port (Table 2).

Most of the Norwegian Sea, exceeding 100 m in depth, was ruled out by the feasibility study (Fig. 3). Low SST and CHL concentration were also observed driving the SI down (Figs. 2a, c and 6). Currently, no wind farms are constructed in the area (Fig. 1).

### Climate change

**Fig. 7:**
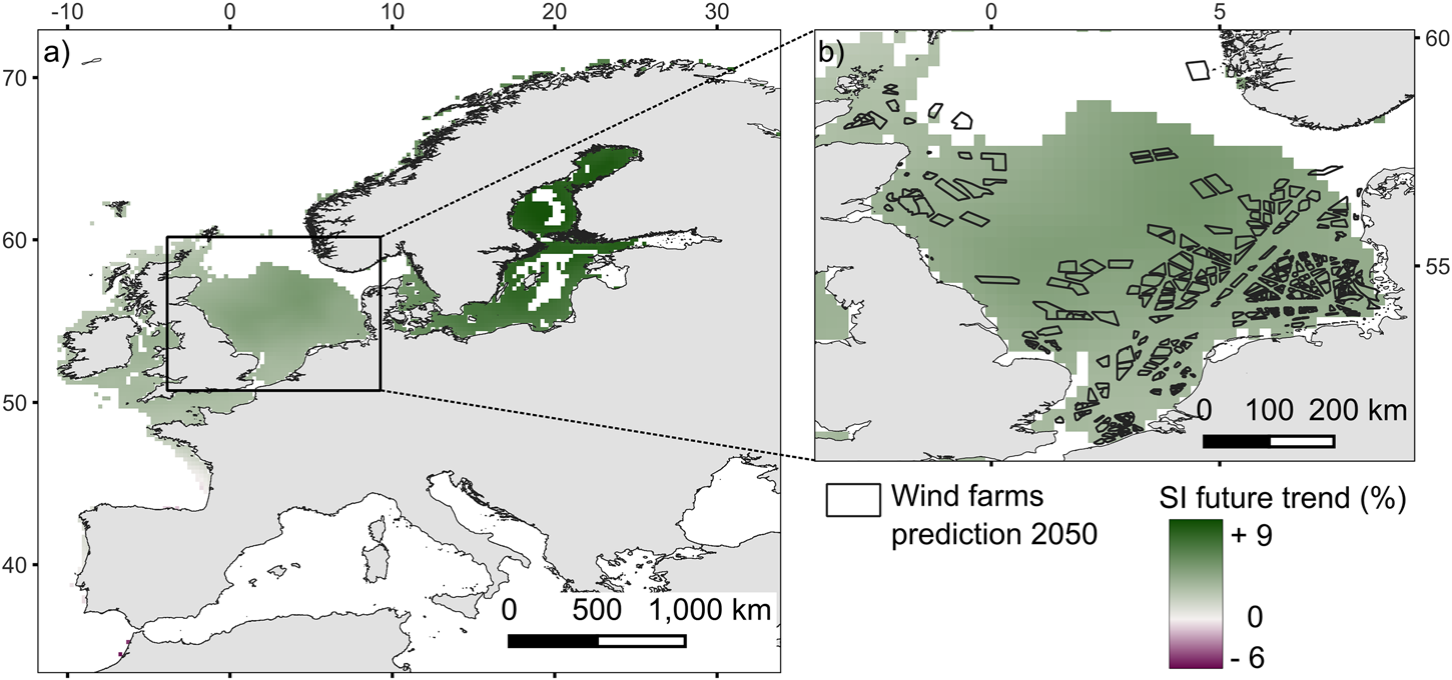
Suitability index (SI) trend for *M. edulis* cultivation under future climate scenarios. **a** SI trend under the SSP5-8.5 scenario (seawater temperature projections). Results are presented as the percentage change in SI between the projected 2040–2050 (Proj) and reference 2010–2020 (Ref) scenarios: 100 × (Proj − Ref)/Ref. **b** Offshore wind farm prediction in the North Sea by 2050 (data from ref. ^29^).

The trend observed from the SI computed using Bio-Oracle 2010–2020 and 2040–2050 SST data revealed a slight fluctuation of the SI (from −6 % to +9 %). An increase in SI for the northern half of Europe (above latitude 47°) and a decrease in the southern half (below latitude 46°) was observed (Fig. 7a). The areas with the largest increase in SI were the ones with the lowest SI initially, as shown in Fig. 5. This could depict a homogenisation of suitability for mussel cultivation in the North Sea. The only area with a very high SI also increasing the most by 2050 is the German Bight, which potentially would present ideal conditions for mussel growth by mid-century.

For the potential 345 farms predicted in the North Sea by Waldman et al.^29^ for 2050, only two did not overlap with a feasible area. The OSW farms predicted by 2050 in the North Sea might experience a slight increase in suitability (Fig. 7b). As most of the OSW farm sites in operation or planned in the future are in the northern half of Europe (Fig. 1), most of them might experience an increase in SI, especially for the farms to be built in the Baltic Sea where the SI could increase up to + 9% due to the SST increasing and getting closer to *M. edulis* optimal temperature (Fig. 7).

## Discussion

This study identified feasible offshore areas for *M. edulis* cultivation and refined these feasible areas by attributing a suitability index. Most of the North Sea, the Irish Sea, the English Channel, and the western coast of France were found to be highly to very highly suitable for *M. edulis* cultivation. Nearshore, these regions corresponded to where mussel aquaculture has been established for a long time^8^. For the North Sea, the same pattern of high suitability in the southern part, decreasing toward the North was found in Benassai et al.^44^, by using different criteria such as wind speed, water depth, SST-anomalies and CHL-anomalies. In the southern part of Europe, only the North Western Iberian and Portuguese coastlines were found feasible, as well as presenting a very high SI. This is likely to be due to the upwelling occurring in the area, bringing colder water and increasing phytoplankton biomass at the surface^60^.

Most of the OSW farms either planned, approved, under construction or in operation overlapped with a feasible area, and presented a high SI. This is aligned with the report of *M. edulis* colonising wind farms, oil and gas platforms, and buoys across these latitudes, often at very high densities^61–64^. As for the Baltic Sea, the feasible areas presented a lower SI. These areas correspond to where the main mussel species colonising the structures is *M. trossulus*, more adapted than *M. edulis* to the environmental conditions^65^.

By 2050, we showed that many OSW projects might overlap with areas that keep a high index or become even more suitable for aquaculture. In this regard, the impact of warming seawater (as a proxy for climate change) was found to increase opportunities for co-location with the OSW industry due to slightly better conditions for growth, in line with the increase in wind farm density.

It is important to stress that this study remains hypothetical. While some OSW farms showed a high SI, were located near ports and were generally accessible for most of the year, this does not automatically imply that aquaculture infrastructure should be established at these sites. Additional environmental factors, such as the presence of protected species and the ecological carrying capacity, must also be taken into account. Furthermore, the feasibility of co-locating aquaculture will depend on the willingness of OSW farm operators to share space and accommodate additional users.

The SI evolution for *M. edulis* under the SSP5-8.5 climate change scenario followed a latitudinal gradient. This type of linear gradient is well known and was also found in the past as a response to climate change, by considering multiple environmental variables predicted under RCP scenarios, applied to dynamic modelling^66–68^. Thermal conditions constitute the primary determinant of ectothermic species’ geographic distribution along latitudinal gradients and their vulnerability to climatic fluctuations^69^. In the framework of this study, with warming waters, the yearly averaged SST in the northern half of Europe (from latitude 46°) gets closer to the optimal seawater temperature for growing *M. edulis* (15.8 °C^70^) while the southern half gets too warm. Yet, the impact of climate change on mussels has not always been observed on a latitudinal gradient, as natural variability can lead to cumulative effects and affect communities at a very local scale^71^. At a population level, an increase in seawater temperature can also lead to a higher mortality rate due to thermal stress^66,72^. Also, a general decrease in population biomass can be expected due to a change in the spatial and temporal scale of recruitment^67,68^.

Overall, the effect of climate change is difficult to predict, even at the individual species level. With the rise of temperature, climate change is likely to lead to lower dissolved oxygen and a lower pH of the water, creating a cumulative impact on the mussel development^10,73–75^. From an aquaculture perspective, deoxygenation is linked to a lower condition index (meat-shell ratio) in mussels, potentially leading to unhealthy production^13^. Altogether, offshore cultivation on suspended lines, a few metres deep (to avoid daily temperature fluctuations) and off-bottom (to limit predation), would be recommended to limit the negative impacts from climate change as opposed to other types of cultivation methods^66,71,72^.

The heat spike definition used in this study was designed for *M. edulis* to underpin the direct impact of warming seawater on its physiology. This allowed a species-specific definition built from in-situ observation and laboratory experiment. It is important to stress that other species, for instance, *M. galloprovincialis*, cultivated in the Mediterranean Sea, might respond differently to heat spikes and temperature variations. For the bigger picture, marine heatwaves (MHWs) are described as 5 days above the 90th percentile of a 30-year SST historical baseline period^76^. This MHW definition is tailored toward ecosystem dynamics rather than specific species responses to increased temperatures, and was therefore not used in this study. Hence, because of climate change, MHWs will happen more often^77,78^, widely impacting marine ecosystems^79,80^. Examples of MHWs were already reported to decrease *M. galloprovincialis* aquaculture performance in Greece^11^ and lead to pathogen breakouts decimating stocks of Pacific oyster in Australia^81^.

The succession of feasibility and suitability analysis enabled more realistic results. For instance, some locations could have received a very high SI score even if they are realistically unfeasible for mussel aquaculture because of deep waters, but also the occurrence of extreme temperature events increasing the risk of mortalities. Indeed, as the SI model relies on how each factor scores on average over the studied period, heat spike episodes might have gone unnoticed, while being detrimental to mussels’ viability. Therefore, the feasibility analysis was found to be efficient in dealing with ‘critical’ criteria, such as heat spike, as well as technical factors like bathymetry and current speed. As technical requirements may be subject to discussion depending on the budget of offshore aquaculture projects or technological advances, the full performance of the SI model across Europe, without any feasibility mask applied, can be found as supplementary material.

The ranking of factors was done as objectively as possible, following the analytical hierarchy process (AHP) method. Nonetheless, the weight from one factor to another depends on the background and point of view of each scientist involved in the ranking. Thus, the model output highly depends on the method used, the criteria selection and the data resolution. For instance, in English waters, the suitability model developed by the Marine Management Organisation (MMO)^18^ scored the lowest in the Channel and the Irish Sea, while it scored the highest in our study. Also, their suitability study scored the highest in the south-west of England, while our method ruled out this area, judged non-feasible due to deep water. These results mainly differ due to the type of factors studied and the SMCE method used, with a Boolean approach in MMO^18^ and adopting a combination of Booleans and a fuzzy method in this study. This suggests that SMCE results should be handled with caution, as they typically address a distinct question with specific requirements. Depending on the question the SMCE is meant to answer, other variables can be considered, such as shipping lanes, protected areas, or underwater cables^82^. Adding more layers to the SMCE can help with final decision-making but can be quite restrictive in exploratory phases.

Another point of consideration when carrying out a SMCE is the spatial and temporal resolution of the environmental data used. Due to the temporal resolution of Bio-Oracle’s SST data (average SST over a decade), heat spikes could not be studied for the 2040–2050 period, and therefore, the feasibility mask was not updated. Yet, it is expected that extreme events of high air and sea temperature will happen more frequently and affect new regions around the globe^83^. Likely, in some areas experiencing an increase in SI in the projected future, mussel aquaculture would not be feasible because of heat spikes. Spatial resolution is also crucial, and requires caution when interpreting results, especially the effect of climate change using coarse resolution climate models^84^. While coarse-resolution satellite imagery and models provide valuable preliminary insights for site assessment, in-situ surveys become essential for final site selection^85^.

The effect of environmental changes can also affect populations differently. Indeed, salinity could be seen as the most important parameter for mussels’ development in the Baltic, as suggested by von Thenen et al.^82^. In the same way, mussel communities in the Baltic Sea might not respond to heat spikes and climate change in the same way as communities off Portuguese coasts. Giomi et al.^86^ demonstrated the importance of considering the thermal history of a population to predict more accurately the physiological response of individuals to climate change. They questioned the role of phenotypic plasticity and adaptation of the organisms in our capacity to predict the effects of climate change on individuals at a global scale (as a comparison to local/community scale). Therefore, it is difficult to predict individual and population responses to climate change. Nevertheless, climate scenario models offered valuable perspectives on the potential spatial patterns of *M. edulis* responses to climate change over the coming decades.

The present research studied the suitability for mussel aquaculture offshore from technical and biological points of view. Socio-economic aspects, administrative specifications, and farmers’ requirements need to be considered to be able to move on to the next stage of potential offshore aquaculture projects^43^. On that, requirements from the OSW and aquaculture sectors need to be assessed and are likely to differ between countries. Thus, this study remains hypothetical and can only be used as a preliminary study to start identifying potentially suitable areas for offshore aquaculture.

Moving mussel aquaculture offshore includes higher costs and uncertainties, heightened by the unpredictable impacts of climate change. Co-location with the OSW industry could help decrease the risks. Operating wind farms could help conduct finer-scale oceanographic sampling to draw a more precise picture of the environmental characteristics of the area, thereby better predicting mussel development and the consequences of climate change. Under potential multi-use projects, shared costs, infrastructure and workforce could help make cultivation even more economically viable^41,42^ as well as environmentally and socially sustainable^87^.

The studied technical offshore aquaculture components, regardless of potential co-existence with the wind industry, still need to be assessed. Especially when moving on to a finer selection, other layers of requirements can be looked at. For instance, seabed type will matter for mooring lines, finding hard and coarse sediment more suitable than fine sand^18^. From a biological perspective, it would be important to assess the area’s capacity for spat collection. Indeed, suitable locations must provide the right conditions (such as appropriate temperature and food availability) for each life stage, as these needs can vary throughout the life cycle of the mussel^13^. This assessment would help determine the most suitable cultivation method, whether relying on natural spat collection or using lines that were pre-colonised in a hatchery, the latter being a lower risk but higher cost option. Some other information has proven difficult to access and rely on in global-scale models. For instance, dissolved O_2_ and pH are important components of bivalve larvae’ survival rate and mussel health. These are often studied together because of their common interaction, which makes it difficult to model the physiological responses of mussels mathematically^73,88^. In addition, no O_2_ or pH datasets were considered relevant in terms of spatial and temporal resolution for this study; thus, these parameters were not used. Nevertheless, these data can be acquired with accuracy via in-situ measurement to help the last stages of site selection.

*M. edulis* is not the only commercially important seafood cultivated in Europe. Based on this work, other bivalves might be considered for offshore aquaculture in areas that were found to be too warm, as some species seemed more resilient to heat stress^89^. For instance, this study did not consider *M. galloprovincialis*, cultivated in the Mediterranean Sea, more resilient to higher temperatures than *M. edulis*^90^. In a general manner, the type of species grown and the site selection need to be done on a fine scale for optimal conditions and aligned with each country’s interests.

As stated previously, the level of co-existence between mussel aquaculture and the OSW industry would need to be decided first. If co-location is agreed on, both industries would share the same area at the same time. If a multi-use project is agreed on, the industries would also share services and infrastructures^32^. For instance, both industries would share transport, and a multi-use platform would allow wind harvesting and mussel cultivation at the same time. This can have a double-edged effect as multi-use entails synergy with mutual benefits for both industries, but also cumulative impacts and therefore the emergence of new conflicts^31^. The ecological carrying capacity of mussel aquaculture was described by Byron et al.^91^, but the cumulative effect with OSW, especially the dense mussel growth already present on the offshore structures, is yet to be described. From that statement, an environmental impact assessment of the cumulative impact of aquaculture and wind farms would need to be carried out, targeting these new conflicts, and thus, for every level of co-existence.

The potential effects of OSW farms on mussel growth still need to be assessed. On the one hand, an increase in phytoplankton concentration has been observed in the vicinity of wind turbines due to the upward transport of nutrients from bottom to surface waters^92^, thus potentially favouring mussel growth. On the other hand, the cultivated mussels may compete for food with the mussels colonising offshore underwater structures^93^.

OSW farms were shown to produce sediment wakes downstream of the bottom-fixed structures^94,95^. These wakes increase suspended sediment toward the surface, and the impact on filter feeders cultivated nearby is yet to be known. Also, turbines’ foundations are protected using coatings containing metal pollutants such as Cd, Pb, and Zn. The corrosion of this coating led to some metal elements being found in excess in the surrounding sediment^96^. So far, no effect on *M. edulis* growth rate was found^97^, nor toxic accumulation in the tissue of mussels growing on the structures^98^. This area of environmental impacts is still understudied, but some industry guidance is possible to minimise the risk of contamination and exposure before food consumption^99^.

This study could be used by decision-makers for potential offshore aquaculture projects. Table 3 lists more detailed feasibility metrics per European country. Also, on a finer scale, the potential option for co-location of mussel aquaculture with the OSW industry can be assessed. Considering the area that could be used for co-location at sea can be difficult to conceive. As an example, mussel farming is an important component of French aquaculture. France was the first global *M. edulis* producer in 2015^100^, however, mussel farms only occupy 7.9 km^2^ of the cadastral plan^101^. Also, France is at the beginning of its OSW industry expansion with 3 farms in production, representing a total of about 270 km^2^ ^102^. Therefore, in a country with significant mussel cultivation activities and low OSW capacity, mussel farms’ surface area only represents 3% of the OSW surface area at sea. From a co-location perspective, and if doubling French mussel farming production was needed, only 3% of the wind farms would be needed, assuming the same yield per m².

**Table 3:** Area and relative area per country’s Exclusive Economic Zone (EEZ) within which *Mytilus edulis* was considered “feasible” and where at least some offshore wind test sites are present in the EEZ

Country	Area in km² (relative area, %)	Country	Area in km^2^ (relative area, %)
Belgium	3100 (88%)	Lithuania	6900 (100%)
Denmark	74,600 (72%)	Netherlands	58,700 (91%)
Estonia	20,400 (56%)	Poland	29,800 (97%)
Finland	64,500 (79%)	Portugal	15,600 (5%)
France	79,500 (23%)	Spain	5600 (1%)
Germany	49,400 (87%)	Sweden	124,700 (80%)
Ireland	123,500 (29%)	United Kingdom	363,600 (50%)
Latvia	15,600 (55%)		

Unlisted countries show that no feasible area was identified in the EEZ.

Yet, the slow implementation of co-location projects and multi-use platforms not only depends on the industries’ technical challenges and cooperation but also on the absence of a regulatory framework, which is a significant brake to offshore aquaculture^103^. Uncertainties around risk assessment and insurance policies are also current concerns for potential stakeholders^37^.

## Methods

In this study, two SMCE methods were used to answer different questions: (i) where is mussel cultivation feasible, and (ii) how suitable is mussel cultivation in feasible areas? The first was answered using the Boolean method (using feasibility thresholds), while the second was answered using fuzzy logic (fitting mathematical models simulating mussel development), more suited for a gradient response^45^.

### Spatial data layers

The E.U. Copernicus Marine Service Information (CMEMS) provides a wide range of global environmental data from modelling, in situ and satellite observations. The environmental factors considered in this study were the SST, surface CHL concentration, SSS, surface SPM concentration, current speed and wave height. These were downloaded from 01/01/2019 to 31/12/2023 to allow a 5-year time series.

Bathymetry data were downloaded from GEBCO (https://betadownload.gebco.net/), and human activities (main ports and OSW activities) were downloaded from EMODnet (https://emodnet.ec.europa.eu/).

To allow the computation of different spatial data resolutions, the bathymetry and current speed data were rescaled on the SST layer resolution of 0.05**°** (approx. 4 km) during the feasibility analysis. Then, in the suitability analysis, CHL and SPM data were rescaled on a 0.25 × 0.25**°** grid resolution (approx. 20 km) corresponding to the SST and SSS layer resolution.

Bio-Oracle (https://www.bio-oracle.org/) was designed for mapping species’ habitat suitability nowadays and in the future^104^, making it a suitable tool for this study. The mean SST from scenario SSP5-8.5 were acquired for the decade 2040–2050 on a 0.05 × 0.05 degree grid resolution. Present-day conditions (decade 2010–2020) were also acquired to allow consistency in spatiotemporal resolution and processing for present-future comparison.

The data used in this study are summarised in Table 4.

**Table 4:** Spatial data layers

Data type	Service	Variables	Period	Spatial resolution	Source
Bathymetry	GEBCO	Ocean depth (m)	2024	400 m	https://betadownload.gebco.net/
Human activities	EMODnet	OSW farms, main transport ports	2024	-	https://emodnet.ec.europa.eu/
SST	CMEMS	Sea surface temperature	Daily 2019–2023	0.25°	10.48670/moi-00165
SST	Bio-Oracle	Mean sea surface temperature	10-year 2010–2020	0.05°	https://www.bio-oracle.org/
SST	Bio-Oracle	Mean sea surface temperature	10-year 2040–2050	0.05°	https://www.bio-oracle.org/
CHL	CMEMS	Mass concentration of chlorophyll a	Gap-free daily 2019–2023	0.05°	10.48670/moi-00281
SSS	CMEMS	Sea surface salinity	Monthly 2019–2023	0.083°, 0.25°	10.48670/moi-0001610.48670/moi-00024
SPM	CMEMS	Mass concentration of suspended inorganic matter	Monthly 2019–2023	0.05°	10.48670/moi-00281
Current speed	CMEMS	Surface current velocity	Daily 2019–2023	0.25°	10.48670/moi-00024
Wave height	CMEMS	Sea surface significant height	Hourly 2019–2023	0.083°, 0.2°	10.48670/moi-00017, 10.48670/moi-00022

### Feasibility and additional criteria for offshore aquaculture

The feasibility of offshore aquaculture mainly depends on criteria such as water depth, waves, and current regimes^105^. Geographical masks, based on water depth and maximum current speed, were used to select feasible areas where the suitability analysis was pertinent to be performed.

Based on the literature, a water depth range of 5 to 100 m^27^ and a threshold of 1 m s^−1^ daily average^22^ were used in the selection.

Also, a seawater surface temperature mask was added to rule out areas that can be exposed to extreme SST and, therefore, considered too high risk for the cultivation (risk of fatalities in the stock). Based on Kamermans & Saurel^13^ observation on *M. edulis* mortalities with rising water temperatures, this study defines a heat spike as at least three consecutive days with SST above 25 °C. Therefore, an area was considered not feasible in the presence of a single heat spike episode over the 5 studied years.

Finally, the number of OSW farms overlapping the feasible areas for mussel cultivation was recorded.

Some criteria were not kept for the feasibility analysis to allow more flexibility but were kept as spatial data layers to support the analysis. This was the case for wave heights, as their impact can vary according to the type of lines and depth of suspension. As wave height also directly impacts the risk for operators at sea, a metric of “accessibility” was computed as the relative time a site is accessible across the five years studied. A site was judged fully accessible if the wave height was always under 1.5 m across the years (100% meaning a site was accessible all the time). This metric is closely linked to technological improvement and is subject to change in the future as operation vessels are designed for rougher seas.

Finally, sustainable distances to ports found in the literature for offshore aquaculture were found to be too restrictive. This study also assesses potential co-location with the OSW industry, sometimes operating further than 55 nm offshore, which could overcome transport challenges by sharing costs. That said, sharing space and transport would increase the level of synergy between both industries.

Every criterion used in the feasibility analysis (as masking criteria and additional criteria) is presented in Table 5.

**Table 5:** Feasibility analysis criteria and thresholds

Criteria	Threshold	References
Feasibility analysis		
Current speed	< 1 m s^-1^	^22^
Bathymetry	5–100 m	^27^
Occurrence of heat spikes (> 25 °C over 3 consecutive days)	<1	
Additional feasibility criteria		
Accessibility time (%, relative time a site is accessible)	<1.5 m significant wave height	^25^
Wave height (maximum)	<6 m maximum wave height	^22^
Distance to port	25 nm and 55 nm	^27,28^

### Suitability analysis

The environmental factors considered in the suitability analysis: SST, CHL concentration, SSS and SPM concentration, are known to influence mussel physiology and growth rate.

An AHP was used to organise these factors depending on their relative importance. It allows the ranking of factors by their pairwise comparison to derive weights and prioritise them^106,107^. To rank the factors, a team of five scientists with expertise in bivalve physiology, marine ecology and ocean-colour remote sensing scored each pair on a scale of 1 (equal importance) to 9 (highest importance of one factor over the other) regarding their relationship. Following Saaty^108^, the pairwise comparison from the AHP was carried out and gave weights to each factor (Table 6). A Consistency Ratio of 0.058 (<0.1) was obtained, showing good consistency in the judgments of the final consensus^108^.

**Table 6:** Factor weights

Factor	SST	CHL	SSS	SPM
Weight	0.45	0.30	0.16	0.09

After fitting mathematical models for each factor, a score from 0 to 1 was obtained for each factor on a daily (SST, CHL concentration) or monthly resolution (SSS, SPM concentration). Then, the mean of the scores over the 5 years was calculated. These averaged scores were then multiplied by their corresponding weight. Finally, the weighted average scores were summed to yield the suitability index (SI) value, as in Eq. 1:

1 $${SI}=\mathop{\sum }\limits_{i=1}^{n}{w}_{i}\bar{{F}_{i}}$$

where $\bar{{F}_{i}}$ is the suitability score for the factor *i*, and ${w}_{i}$ is its corresponding weight. To map the SI for *M. edulis* cultivation, five classes were used (Table 7).

**Table 7:** Suitability index classification

Suitability level	Very low	Low	Medium	High	Very high
Range	0.0–0.2	0.2–0.4	0.4–0.6	0.6–0.8	0.8–1.0

For each environmental parameter (SST, CHL, SSS, SPM), a mathematical function based on known *M. edulis* responses to environmental variability was retrieved from the literature, summarised in Table 8.

**Table 8:** Environmental factors used in the suitability analysis

Sea surface temperature (SST)			
$$\begin{array}{lll}f\left(SST\right)\\ =\left\{\begin{array}{l}{\left(\frac{{T}_{max}-SST}{{T}_{max}-{T}_{opt}}\right)}^{c({T}_{max}-{T}_{opt})}\cdot {e}^{c\left(SST-{T}_{opt}\right)}\,\,\,\,\,\,\,\,\,\,\,SST < {T}_{max}\\ \,\,\,\,\,\,\,\,\,\,\,\,\,\,\,\,\,\,\,\,\,\,\,\,\,0\qquad\qquad\qquad\qquad\quad\,\,\,\,\,\,\,\,\,\,\,SST\ge {T}_{max}\end{array}\right\}\end{array}$$	${T}_{max}$ = 30 °C ${T}_{{opt}}$ = 15.8 °C $c$ = 0.393		^70^
Sea surface salinity (SSS)			
$y=-9\cdot {10}^{-6}\cdot SS{S}^{4}+6.91\cdot {10}^{-4}\cdot SS{S}^{3}-2.087\cdot {10}^{-2}\cdot SS{S}^{2}+0.296816\cdot SSS-0.875038$			^18,109^
Chlorophyll a concentration (CHL)			
$f=\frac{CHL}{CHL+{X}_{K}}$		${X}_{K}$ = 1.06 (half-saturation coefficient)	^112^
Suspended particulate inorganic matter concentration (SPM)			
$Abs \% =-2\cdot {10}^{-4}\cdot SP{M}^{2}-4.2\cdot {10}^{-3}\cdot SPM+0.8273$			^111^

**Fig. 8:**
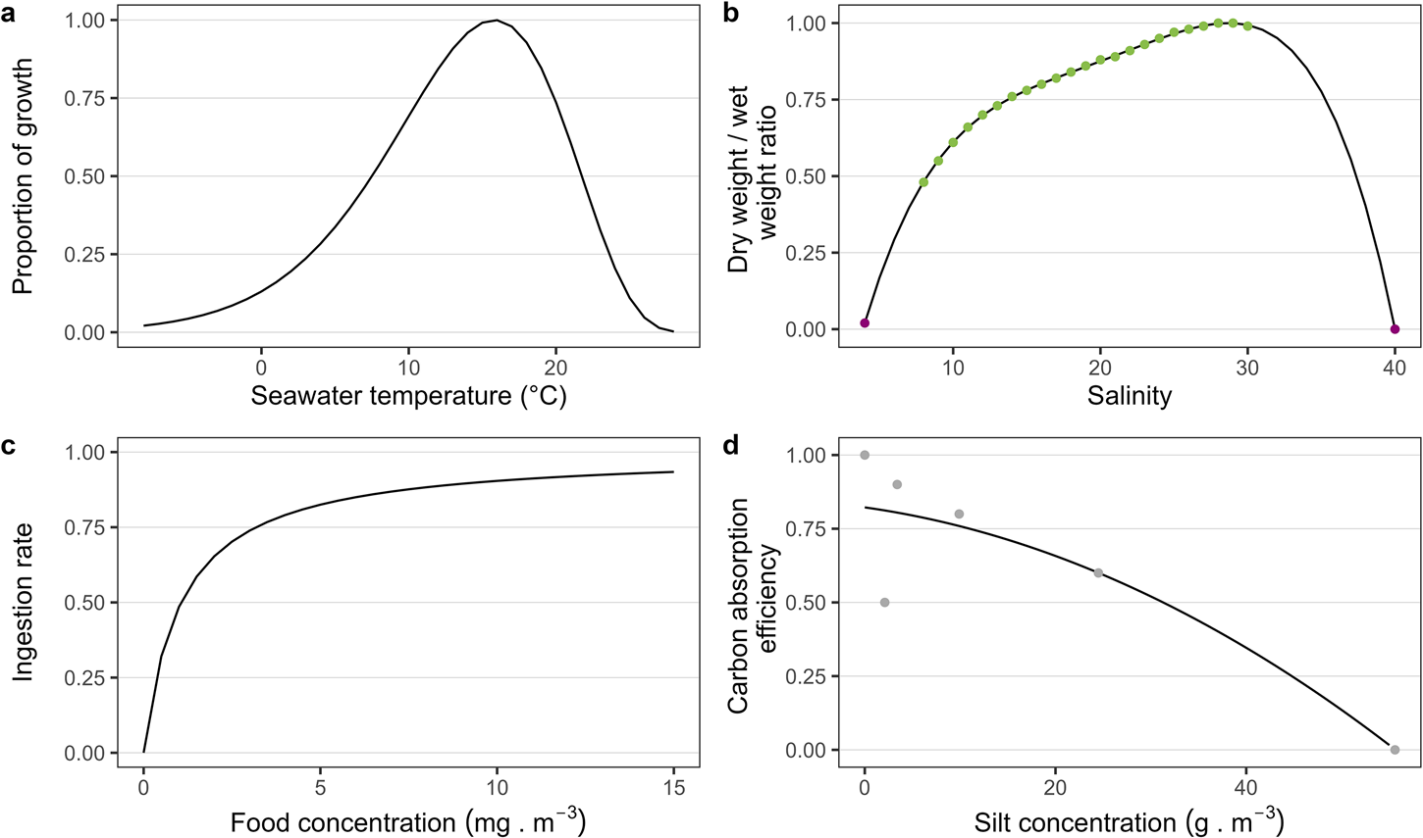
Graphical representation of *M. edulis* physiological responses to environmental factors. Different physiological responses of *M. edulis* are shown: **a** proportion of growth as a function of seawater temperature, **b** dry weight/wet weight ratio as a function of salinity with data from MMO^18^ (purple dots) and Maar et al.^109^ (green dots), **c** ingestion rate as a function of food (CHL) concentration, and **d** carbon absorption efficiency as a function of silt (SPM) concentration with fitted data from Kiørboe et al.^111^.

In the case of SSS, two datasets had to be pulled together in order to cover a larger range of salinity for a European-scale application. Thus, values from the MMO^18^ were added to Maar et al.^109^ data, and a polynomial function was fitted in this new dataset in R^110^. Further, a polynomial function was fitted to the experimental SPM dataset found in Kiørboe et al.^111^. The graphical representation of each mathematical model is presented in Fig. 8. The studied responses were the proportion of maximum growth, ingestion rate, dry weight-wet weight (DW/WW) ratio, or carbon absorption efficiency rate. Each function was standardised from 0 to 1 by subtracting the minimum value and dividing by the range variation^56^. This way, each environmental factor could be expressed as a score depending on *M. edulis* response to its daily or monthly fluctuations.

*M. edulis* is sensitive to environmental changes (Fig. 8). With SST being the top factor identified in the AHP, the impact of increased temperature on the suitability of *M. edulis* cultivation was assessed using the SST climate scenario from the shared socioeconomic pathway (SSP5-8.5). This way, two other SI were computed: present SI and future SI using the 2010–2020 and 2040–2050 mean SST dataset (the other factors were assumed to stay the same). Then, the percentage of change in SI between the projected 2040–2050 and reference 2010–2020 scenarios was obtained to observe the possible evolution of the SI in the future.

## Conclusions

The available space at sea is increasingly limited in Europe. The OSW industry is showing a considerable expansion at sea to meet national commitments to a low-carbon future. The interest in the aquaculture industry to move further offshore has attracted growing interest, as this could increase production significantly, as well as mitigate some impacts of climate change. These ambitious goals cannot be met without concessions and sharing the space with others. This can imply different degrees of co-existence and synergy.

This study provided a general view of potentially feasible (technically) and suitable (biologically) locations for offshore aquaculture of the blue mussel (*Mytilus edulis*), with an insight into potential co-location with the wind industry. Above 1,000,000 km² were found to be technically feasible for offshore aquaculture in Europe. The southern part of the North Sea, as well as the coasts of the United Kingdom, Ireland, France and Portugal were found to be suitable for mussel growth. Most of the OSW farms already constructed in Europe were located in suitable areas for mussel cultivation, highlighting options for co-location. Even more options will likely be available in the future, given the expansion of the OSW sector in the North Sea.

Therefore, this study gives insights for MSP and policy-making on the potential synergy between mussel aquaculture and the energy sector at the European level. The use of a standardised SMCE method also allows the study of different cultivated species and levels of co-existence between the industries.

However, the impact of climate change is difficult to predict, and the higher occurrence of extreme events can be a challenge to offshore aquaculture, bringing uncertainty to the viability of such a project. In addition, a full socio-economic analysis would be necessary to move on to the next stage of MSP. Nevertheless, the shared maritime space is a crucial component in securing global food and energy supplies in response to the growing and emerging challenges driven by climate change and increasing ocean use.

## Supplementary information

Supplementary Information

## Data Availability

The datasets analysed and generated during this study, as well as the codes developed, are available in the GitHub repository: https://github.com/EnoLec/smce_mussel_osw_europe.
