# Supplementary material for: Identifying suitable mussel cultivation sites in European offshore waters—an assessment for co-location with the wind industry
Source: NPJ Ocean Sustain. 2026 Feb 27;5(1):20. doi: 10.1038/s44183-026-00187-0 (PMC13065476; doi:10.1038/s44183-026-00187-0)
Supplement: Supplementary file 1 — Supplementary Information [file 44183_2026_187_MOESM1_ESM.pdf]

Supplementary Materials

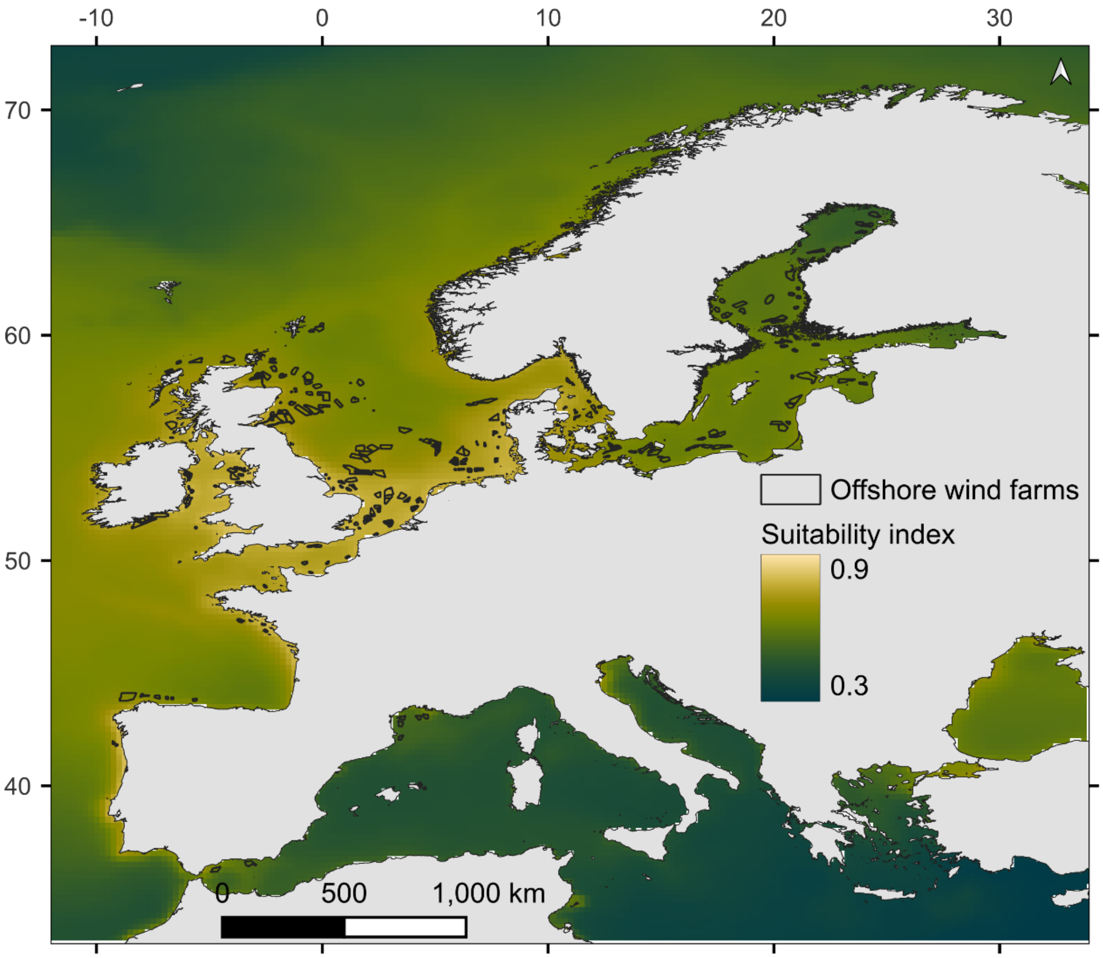

Fig. S1: **Suitability index for Mytilus edulis cultivation in Europe.** The entire study extent is shown (the feasibility mask not applied). Black polygons show offshore wind farms either in production, under construction, approved for construction, or planned.
